# Neuroprotective Effects of *Rosa roxburghii* Tratt Juice Concentrate Powder in Parkinson’s Disease Mice via the PI3K/AKT Signaling Pathway

**DOI:** 10.3390/ph19050711

**Published:** 2026-04-30

**Authors:** Tong Jin, Long Liu, Faguang Kuang, Mingjie Chen, Haonan Chen, Jiapan Deng, Yikai Yang, Baofei Sun, Heng Luo

**Affiliations:** 1 Key Laboratory of Human Brain Bank for Functions and Diseases, Department of Education of Guizhou Province, College of Basic Medical, Guizhou Medical University, Guiyang 561113, China; 2 State Key Laboratory of Discovery and Utilization of Functional Components in Traditional Chinese Medicine, Natural Products Research Center of Guizhou Province, School of Pharmaceutical Sciences, Guizhou Medical University, Guiyang 550014, China

**Keywords:** PD, RRJCP, network pharmacology, PI3K/AKT pathway, mice

## Abstract

**Background**: The absence of disease-modifying treatments for Parkinson’s disease (PD)—a neurodegenerative condition with escalating global incidence—represents a critical unmet medical need. Traditionally utilized for both dietary consumption and medicinal preparations, the fruit derived from *Rosa roxburghii* Tratt demonstrates a remarkably rich profile of biologically active compounds, with flavonoids, triterpenoids, and organic acids representing the predominant classes. Experimental evidence indicates that these compounds elicit robust antioxidative, anti-inflammatory, and neuroprotective effects, making them promising candidates for neurodegenerative disease modulation. This study aimed to systematically evaluate the neuroprotective effects of *Rosa roxburghii* Tratt juice concentrate powder (RRJCP) across the preventive, interventional, and therapeutic stages of PD and to elucidate its underlying molecular mechanisms. **Methods**: *Rosa roxburghii* Tratt juice was subjected to rotary evaporation concentration and vacuum freeze-drying to obtain the juice concentrate powder. C57BL/6 mice were randomly assigned to three main groups (prevention, intervention, and treatment), each containing subgroups including a normal control, an MPTP model group, low-, medium-, and high-dose RRCJP groups (50, 100, and 200 mg/kg), and a positive control Madopar group, totaling 18 subgroups. A chronic MPTP-induced PD mouse model was established. Motor function was assessed via the open field test, pole test, and wire hang test. Substantia nigra neuronal morphology was examined by hematoxylin and eosin staining. The area of tyrosine hydroxylase (TH)-positive regions was measured by immunohistochemistry. The levels of oxidative stress indicators in serum were measured using biochemical kits. Network pharmacology was employed to predict core targets, and the expression of PI3K/AKT pathway and apoptosis-related proteins was determined by Western blotting. **Results**: Compared with the MPTP model group, RRCJP (200 mg/kg) significantly increased the total distance traveled in the open field, shortened the pole climbing time, and improved the wire hang score. It attenuated the morphological disorganization and nuclear pyknosis of substantia nigra neurons, increased the TH-positive area and TH protein expression, reduced serum MDA content, and elevated the activities of SOD and GSH-Px. Network pharmacology analysis indicated that the PI3K/AKT signaling pathway was among the core targets. Western blotting results further showed that the juice concentrate powder upregulated the expression of p-PI3K, p-AKT, and Bcl-2, while downregulating Bax and Cleaved Caspase-3 levels, which was consistent with the network pharmacology prediction. **Conclusions**: RRCJP exerts neuroprotective effects across the preventive, interventional, and therapeutic stages in PD model mice, the mechanisms of which may be associated with activation of the PI3K/AKT signaling pathway, attenuation of oxidative stress, and inhibition of neuronal apoptosis.

## 1. Introduction

In terms of global disease burden, Parkinson disease (PD) occupies the position of the second most frequently encountered neurodegenerative disorder, exceeded in prevalence only by Alzheimer disease, whereas epidemiological statistics reveal a continuous annual rise in documented cases [1]. From a clinical perspective, affected individuals typically present with cardinal motor deficits encompassing tremor at rest, bradykinesia, muscular hypertonia, and impairments in postural stability and gait, accompanied by nonmotor sequelae including hyposmia, disrupted sleep architecture, autonomic failure, and progressive neurocognitive decline [2,3]. Histopathologically, the defining alterations consist of selective attrition among dopaminergic neuronal populations situated in the substantia nigra of the ventral midbrain. This is accompanied by the intracellular accumulation of fibrillar proteinaceous deposits designated as Lewy bodies, the primary biochemical constituent of which is aggregated alpha-synuclein [4]. The pathogenesis of this disease involves multiple mechanisms, including mitochondrial dysfunction, oxidative stress, inflammation, and apoptosis [5,6,7]. Currently, the treatment of Parkinson’s disease primarily relies on pharmacological and surgical approaches. Pharmacologically, dopaminergic medications are used as replacement therapy to supplement the deficiency of dopamine in the brain. However, these methods are still unable to fundamentally halt the progression of the disease [8,9]. The high diagnostic and treatment costs severely impact patients’ quality of life and have significant socioeconomic consequences, making the development of new drugs an urgent medical challenge that needs to be addressed [10,11].

The medicine and food homology (MFH) substances have garnered significant attention in both the food industry and medical research due to their dual nutritional and therapeutic value [12]. Not only do they provide essential daily nutrients, but they also exhibit various biological activities, including anti-fatigue effects, immune regulation, and neuroprotection, demonstrating considerable potential in the prevention and management of chronic diseases. *Rosa roxburghii* Tratt (RRT), a species of the Rosaceae family, is a distinctive plant with both edible and medicinal properties, offering unique nutritional and therapeutic value [13]. As a wild plant unique to the mountainous regions of western China, its fruit is highly sought after by consumers for its high nutritional value and health benefits [14]. The fruit of RRT is rich in functional bioactive components such as polysaccharides, terpenoids, multiple vitamins, flavonoids, superoxide dismutase (SOD), polyphenols, trace elements, and organic acids. It exhibits various biological activities, including anticancer, antioxidant, anti-aging, anti-atherosclerotic, hypoglycemic, immunomodulatory, radioprotective, digestive regulatory, anti-inflammatory, cardioprotective, antibacterial, and gut microbiota modulating effects. As such, it represents an important natural resource with significant nutritional and medicinal value [13,14,15]. Studies have found that in a type 1 diabetic mouse model, RRT juice can improve insulin signaling, promote hepatic glycogen synthesis and glucose transport by activating the PI3K/AKT signaling pathway, thereby effectively lowering blood glucose, regulating blood lipids, and reducing oxidative damage. In a reproductive system injury model, activation of the PI3K/AKT signaling pathway can inhibit testicular cell apoptosis, alleviate oxidative stress and inflammation, and facilitate DNA repair, thereby improving sperm quality and testicular function [16,17]. These two studies collectively reveal the pleiotropic regulatory mechanism of RRT juice based on the PI3K/AKT pathway, providing key mechanistic evidence for its application as a MFH substance in the fields of metabolic and reproductive health. Given that PI3K/AKT signaling is crucial for dopaminergic neuron survival and its dysfunction contributes to PD pathogenesis, RRT juice’s activation of this pathway suggests potential relevance to PD neuroprotection. Furthermore, a specific triterpenoid saponin compound from the fruit of RRT, Kaji-ichigoside F1, has been shown to significantly alleviate NMDA-induced neurotoxicity by modulating the BDNF/Akt/mTOR signaling pathway, demonstrating clear neuroprotective effects in vitro [18]. Therefore, RRT juice may hold potential for protecting dopaminergic neurons and alleviating symptoms in PD. However, its specific mechanisms remain unclear.

This study aimed to investigate the preventive, interventional, and therapeutic effects of RRJCP in an MPTP-induced chronic Parkinson’s disease mouse model, and to elucidate its underlying molecular mechanisms by integrating network pharmacology prediction with experimental validation. The study evaluated the intervention effects of RRJCP across three stages—before, during, and after PD onset—thereby transcending the limitations of previous studies that focused on a single administration time point. The findings revealed that RRJCP exerts multi-target neuroprotective effects by activating the PI3K/AKT signaling pathway and inhibiting oxidative stress and apoptosis. This work provides an experimental basis for the development of safe, orally administrable RRJCP-based health foods or novel herbal medicines as adjunctive interventions for PD, offering the potential to reduce long-term medication costs and improve patients’ quality of life, while also providing a modern scientific interpretation of the Traditional Chinese Medicine principle of “preventive treatment of disease”.

## 2. Results

### 2.1. RRJCP Ameliorates Behavioral Deficits in PD Mice

**Figure 1:**
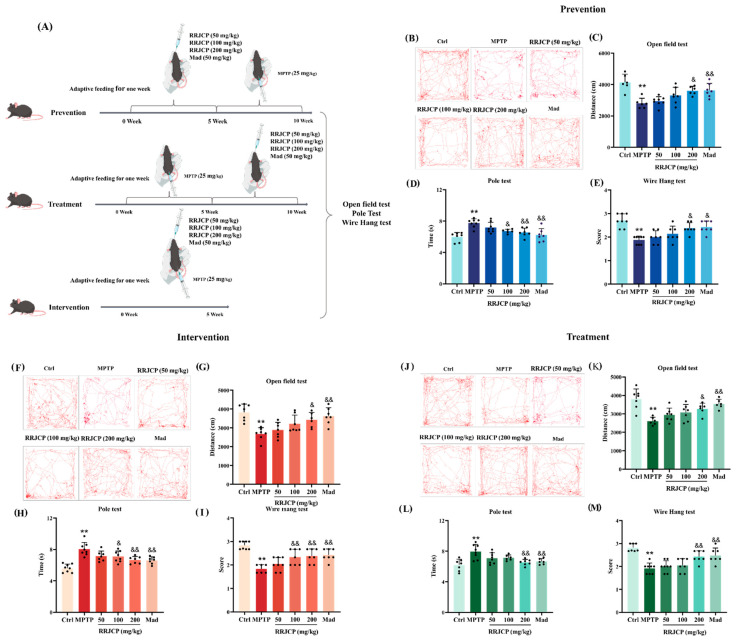
RRJCP can improve behavioral impairments in PD mice. (**A**) Experimental flowchart of the mouse model; (**B**–**E**) Behavioral statistics for the prevention group; (**F**–**I**) Behavioral statistics for the intervention group; (**J**–**M**) Behavioral statistics for the treatment group. Data are presented as the mean ± SD. ** *p* < 0.01 vs. Ctrl; ^&^ *p* < 0.05, ^&&^ *p* < 0.01 vs. MPTP.

Prevention, Intervention, and Treatment groups: Total distance traveled in the open field was significantly reduced (*p* < 0.01), pole climbing time was significantly prolonged (*p* < 0.01), and tail suspension score was significantly decreased (*p* < 0.01) in MPTP model mice relative to the Ctrl group. Conversely, RRJCP 200 mg/kg and Mad significantly reversed these alterations, increasing total distance traveled (*p* < 0.05, *p* < 0.01), shortening pole climbing time (*p* < 0.05, *p* < 0.01), and improving Wire Hang score (*p* < 0.05, *p* < 0.01) compared with the model group (Figure 1B–M).

### 2.2. RRJCP Attenuates MPTP-Induced Damage in the Substantia Nigra of Mice

**Figure 2:**
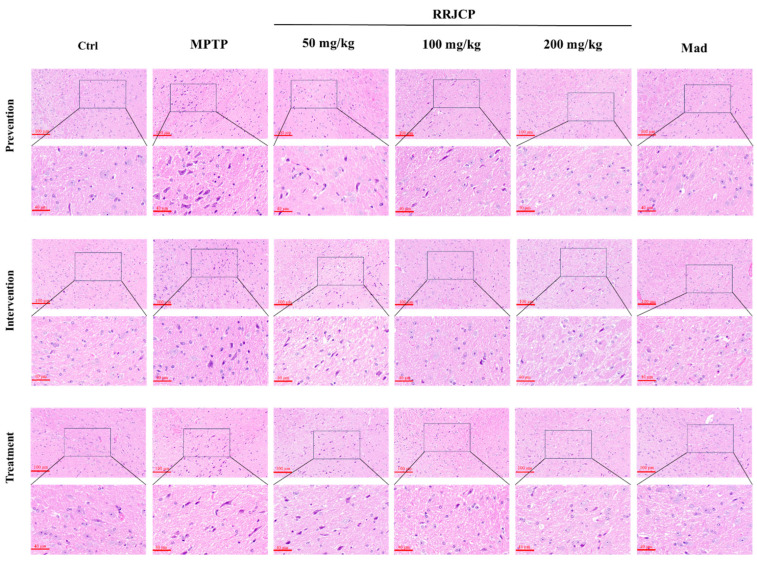
HE of the midbrain substantia nigra in mice. The nuclei are dark blue, and the cytoplasm is pink.

Prevention, Intervention, and Treatment: In the Control groups, neurons within the substantia nigra tissue exhibited regular morphology, a neat and densely packed arrangement, normal shape, relatively large nuclei, clear contours, and uniform staining. In the MPTP groups, the substantia nigra tissue showed disorganized neuronal arrangement, a reduction in cell number, and extensive neuronal degeneration and death. In the RRJCP 200 mg/kg and Mad groups, the morphology of substantia nigra neurons appeared largely normal, with large nuclei and clear contours (Figure 2).

### 2.3. RRJCP Attenuates TH-Positive Neuron Loss in PD Mice

**Figure 3:**
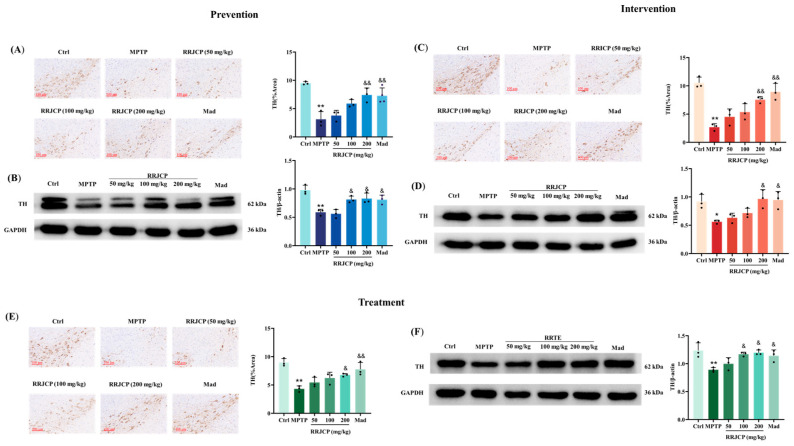
Immunohistochemical staining and Western blotting of TH in the substantia nigra of mice from each group. (**A**,**B**) Statistical analysis of TH immunohistochemistry positive area rate and Western blotting in the prevention group. (**C**,**D**) Statistical analysis of TH immunohistochemistry positive area rate and Western blotting in the intervention group; (**E**,**F**) Statistical analysis of TH immunohistochemistry positive area rate and Western blotting in the treatment group. Data are presented as the mean ± SD. * *p* < 0.05, ** *p* < 0.01 vs. Control; ^&^ *p* < 0.05, ^&&^ *p* < 0.01 vs. MPTP.

Prevention, Intervention and Treatment: TH immunoreactive area in midbrain substantia nigra was significantly reduced (*p* < 0.05, *p* < 0.01) in MPTP model mice compared with Ctrl, as demonstrated by immunohistochemistry. Relative to MPTP, RRJCP 200 mg/kg and Mad significantly increased TH immunoreactive area (*p* < 0.05, *p* < 0.01) (Figure 3A,C,E). TH protein expression in substantia nigra was significantly decreased (*p* < 0.05, *p* < 0.01) in MPTP group versus Ctrl per Western blotting. Conversely, RRJCP 200 mg/kg and Mad significantly elevated TH protein expression in substantia nigra relative to MPTP (*p* < 0.05, *p* < 0.01) (Figure 3B,D,F).

### 2.4. RRJCP Alleviates Oxidative Stress in MPTP-Induced PD Mice

**Figure 4:**
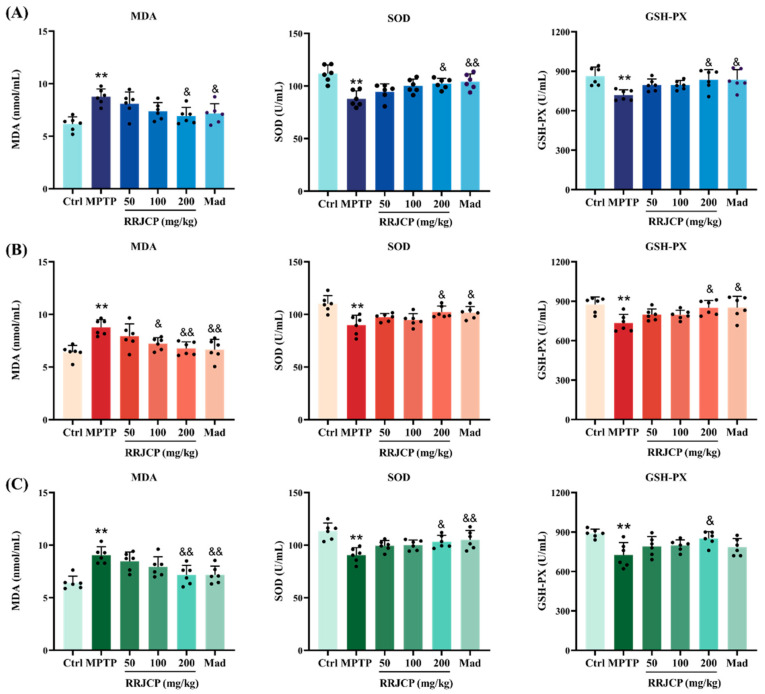
RRJCP alleviates oxidative stress in PD mice. (**A**) Oxidative Stress Statistics for the Prevention Group; (**B**) Oxidative Stress Statistics for the intervention Group; (**C**) Oxidative Stress Statistics for the treatment Group. Data are presented as the mean ± SD. ** *p* < 0.01 vs. Ctrl. ^&^ *p* < 0.05, ^&&^ *p* < 0.01 vs. MPTP.

Prevention, Intervention and Treatment: Serum SOD and GSH-PX activities were significantly decreased (*p* < 0.05, *p* < 0.01), and MDA content was significantly increased (*p* < 0.01), in MPTP group relative to Ctrl. Relative to MPTP, RRJCP 200 mg/kg and Mad significantly restored serum SOD and GSH-PX activities (*p* < 0.05, *p* < 0.01). Conversely, these treatments significantly diminished serum MDA content relative to MPTP (*p* < 0.05, *p* < 0.01) (Figure 4A–C).

### 2.5. LC-MS/MS Analysis of RRCJP

**Figure 5:**
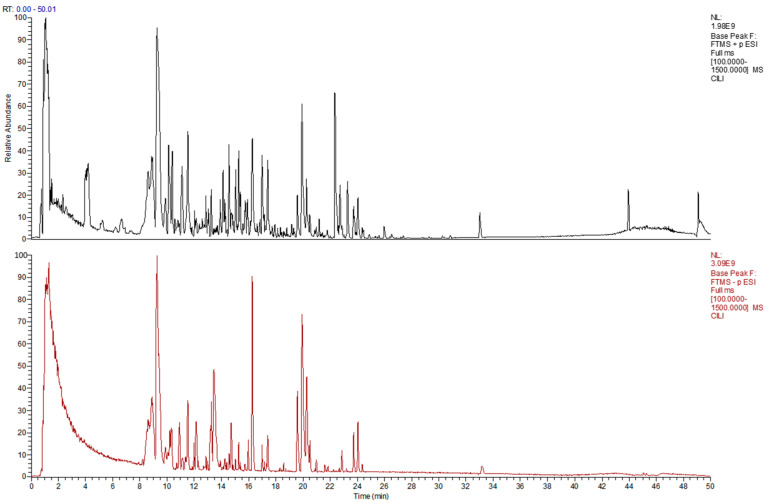
Total ion chromatogram.

The total ion chromatogram (TIC) of RRJCP (Figure 5), acquired in positive/negative ion switching mode, exhibited well-resolved and symmetrical peaks, demonstrating effective chromatographic separation of the major chemical constituents in RRJCP (Table 1). By matching accurate mass, retention time, and MS/MS fragmentation patterns against spectral libraries, a total of 25 compounds were identified (Table 1). These compounds belong to diverse structural classes, including flavonoids (e.g., (+)-Catechin hydrate, isoquercitrin), phenolic acids (e.g., ellagic acid, ferulic acid), triterpenoids (e.g., rosamultin, asiatic acid, oleanonic acid), organic acids (e.g., citric acid, quinic acid), and amino acids. Notably, (+)-Catechin hydrate, procyanidin B1, and ellagic acid exhibited relatively high peak areas, representing characteristic constituents of RRJCP consistent with previous reports on *Rosa roxburghii* Tratt fruit.

### 2.6. Screening of RRJCP Active Ingredients, Target Prediction, and Screening for PD Targets

**Figure 6:**
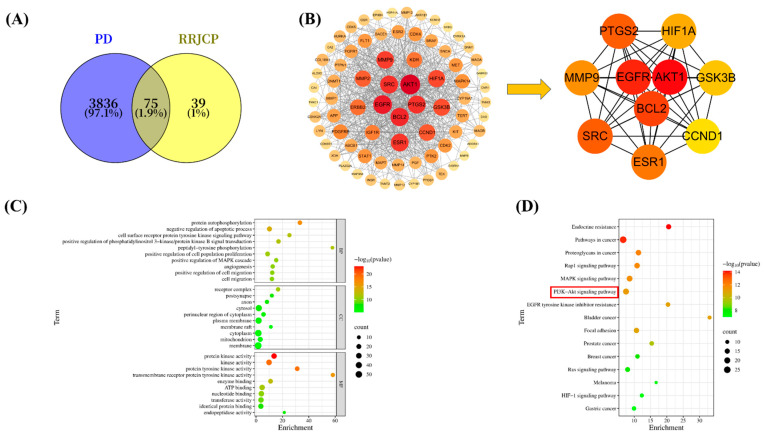
Network pharmacology analysis. (**A**) Intersection targets of PD and RRJCP; (**B**) PPI network of potential targets for RRJCP intervention in PD; (**C**) GO analysis; (**D**) KEGG analysis, The red box indicates that this pathway is one of the key pathways.

Based on the 25 chemical components identified in RRJCP (as shown in Table 1), active compounds were screened using the TCMSP database. The targets of these active compounds were subsequently predicted using the Swiss Target Prediction database, yielding a total of 114 RRJCP-related targets. For PD-associated targets, a search was conducted in the GeneCards and OMIM databases using “Parkinson’s disease” as the keyword. After merging the results and removing duplicates, a total of 3911 targets closely related to PD were identified. A Venn diagram analysis was then performed on the 114 RRJCP targets and the 3911 PD targets using the Microbioinformatics platform (Figure 6A), revealing 75 overlapping targets. These intersecting targets are considered potential candidates through which RRJCP may exert protective effects against Parkinson’s disease.

### 2.7. Construction of the PPI Network and Identification of Core Targets

The 75 overlapping genes were imported into the STRING database to obtain a TSV format file containing protein–protein interaction relationships. This file was then imported into Cytoscape 3.10.3 to generate a visual network diagram. Using Cytoscape 3.10.3 for analysis and screening based on the Degree value, the top 10 targets ranked were: AKT1, EGFR, BCL2, PTGS2, SRC, ESR1, MMP9, HIF1A, GSK3B, CCND1. This suggests that these targets may play key roles in the pathological process of RRJCP in preventing and treating PD (Figure 6B).

### 2.8. GO Enrichment Analysis and KEGG Pathway Analysis

The overlapping genes were submitted to the DAVID database for GO enrichment analysis and KEGG pathway analysis. The GO enrichment analysis primarily encompasses BP, CC, and MF, yielding 352, 78, and 108 enriched terms (Figure 6C). At the Biological Process level, the key targets are predominantly associated with protein autophosphorylation, negative regulation of apoptotic process, cell surface receptor protein tyrosine kinase signaling pathway, positive regulation of phosphatidylinositol 3-kinase/protein kinase B signal transduction, peptidyl-tyrosine phosphorylation, positive regulation of cell population proliferation, positive regulation of MAPK cascade angiogenesis, positive regulation of cell migration, cell migration. At the Cellular Component level, these targets are mainly localized to the receptor complex, postsynapse, axon, cytosol, perinuclear region of cytoplasm, plasma membrane, membrane raft, cytoplasm, mitochondrion, and membrane. At the Molecular Function level, they mainly participate in protein kinase activity, kinase activity, protein tyrosine kinase activity, transmembrane receptor protein tyrosine kinase activity, enzyme binding, ATP binding, nucleotide binding, transferase activity, identical protein binding, and endopeptidase activity. The KEGG pathway analysis identified 110 pathways. Among the top 15 ranked KEGG pathways, major ones involve the PI3K-Akt signaling pathway, among others (Figure 6D).

### 2.9. RRJCP Activates the PI3K/AKT Signaling Pathway While Concurrently Modulating the Expression of Apoptosis-Related Proteins

**Figure 7:**
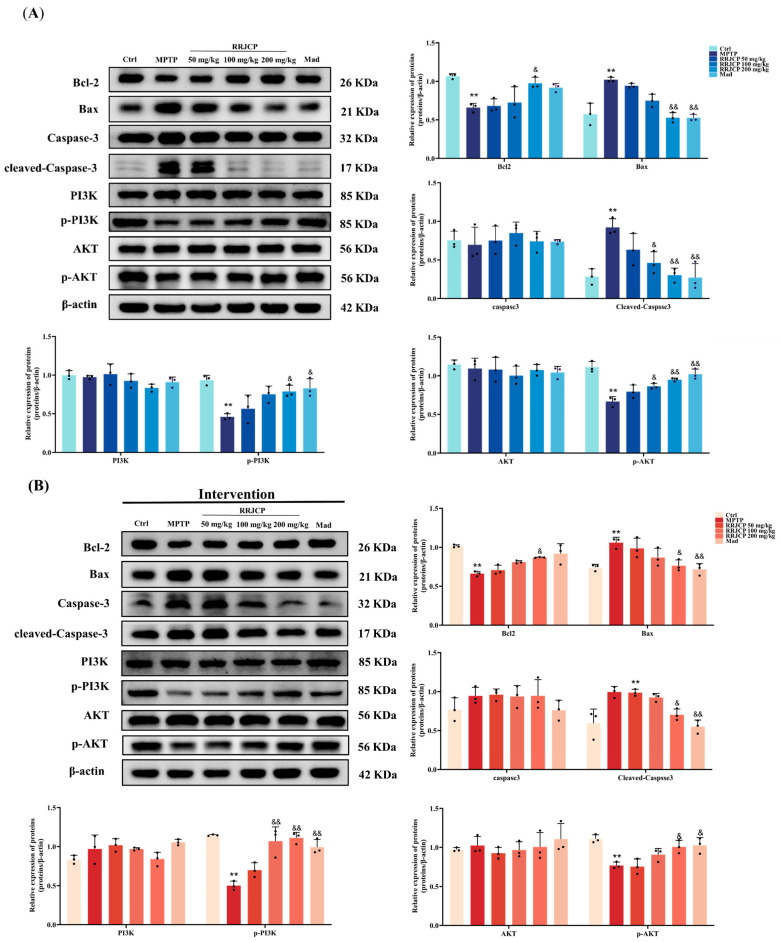

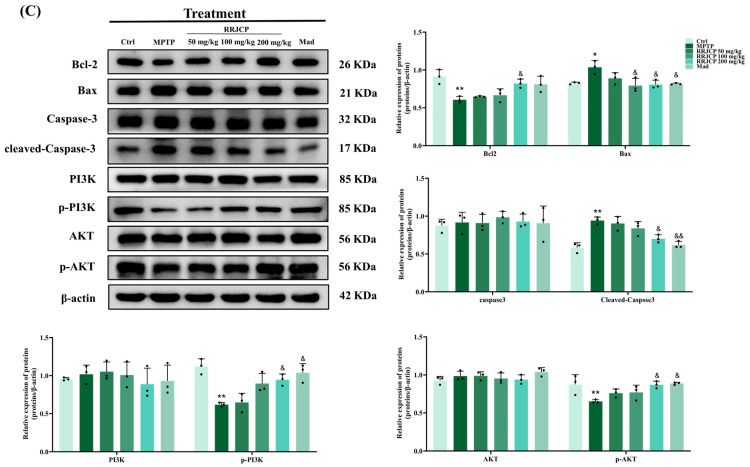
Expression changes of PI3K/AKT pathway and apoptosis-related proteins in the mouse substantia nigra. (**A**) Prevention group; (**B**) Intervention group; (**C**) Treatment group. Data are presented as the mean ± SD. * *p* < 0.01, ** *p* < 0.01 vs. Ctrl. ^&^ *p* < 0.05, ^&&^ *p* < 0.01 vs. MPTP.

This study was undertaken to elucidate the molecular basis underlying the ameliorative effects of RRJCP on PD. Prior network pharmacology predictions indicated that RRJCP may exert its beneficial actions via activation of the PI3K/AKT signaling cascade and modulation of apoptotic processes. Accordingly, immunoblotting was employed to assess the expression alterations of pivotal proteins within the PI3K/AKT pathway and apoptosis-associated factors in the substantia nigra of mice across distinct experimental cohorts. The findings revealed that in the prevention, intervention, and treatment phases, the MPTP-lesioned group exhibited marked reductions in the ratios of phosphorylated PI3K (p-PI3K) and phosphorylated AKT (p-AKT) relative to the control cohort (*p* < 0.05, *p* < 0.01), accompanied by pronounced elevations in the ratios of Bax and cleaved caspase-3 (*p* < 0.01). In contrast, administration of RRJCP at 200 mg/kg, as well as the positive control Madopar, led to significant restoration of p-PI3K, p-AKT, and Bcl-2 ratios (*p* < 0.05, *p* < 0.01), while concurrently suppressing the levels of Bax and activated caspase-3 compared to the MPTP group (*p* < 0.05, *p* < 0.01) (Figure 7A–C).

## 3. Discussion

Neurodegenerative diseases are becoming an increasingly pressing global health challenge, particularly in the context of an aging population. Among them, PD is one of the most common and severe [25]. Although advances have been achieved in elucidating the underlying pathophysiology and molecular pathways of the disease, existing therapeutic approaches are predominantly palliative in nature and do not halt or reverse the core degenerative process. This gap in disease-modifying interventions highlights the pressing demand for innovative and efficacious treatment modalities. Bioactive constituents obtained from natural origins, particularly those sourced from edible plants, are increasingly regarded as attractive therapeutic avenues for combating neurodegenerative conditions [26]. Studies have demonstrated that RRJCP is rich in various natural products and nutrients, including organic acids, phenolic compounds, flavonoids, and terpenoids, and exhibits strong free radical scavenging and anti-inflammatory effects [27]. This investigation systematically assessed the neuroprotective capacity of RRJCP across three distinct temporal windows—disease prevention, progression intervention, and post-onset treatment—utilizing a chronic MPTP-induced mouse model of Parkinson’s disease. The findings demonstrate that RRJCP administration markedly alleviated motor behavioral deficits, substantially attenuated the degeneration and depletion of dopaminergic neurons within the substantia nigra, and concurrently suppressed oxidative stress responses while impeding apoptotic cascades. Further mechanistic investigation revealed that the observed neuroprotection was closely associated with the activation of the PI3K/AKT signal transduction axis. Collectively, this work not only provides experimental evidence for the multi-target neuroprotective attributes of RRJCP but also offers novel mechanistic insights into how medicinal-edible homologous compounds regulate the prevention and management of neurodegenerative disorders.

Behavioral improvement is a direct reflection of functional recovery. The open field test, pole test, and Wire Hang test evaluated the motor functions of the mice in terms of autonomous exploration, motor coordination, and muscle strength/endurance, respectively [28,29]. In this study, the open field test, pole test, and wire hang test were employed to evaluate motor function from the perspectives of spontaneous locomotor activity, motor coordination, and muscle strength/endurance, respectively. The results demonstrated that RRJCP resulted in comprehensive improvement across all behavioral assessments: the increased total distance traveled in the open field reflected enhanced exploratory behavior, whereas improvements in the pole and wire hang tests more specifically indicated the normalization of coordination within the cortical-basal ganglia-spinal motor pathway and muscle tone. Notably, high-dose RRJCP (200 mg/kg) significantly reversed MPTP-induced behavioral deficits irrespective of whether administration occurred during the preventive, interventional, or therapeutic phases, suggesting that its neuroprotective efficacy is not confined to a specific intervention time window. Nevertheless, cross-sectional comparison of the effects elicited by the medium dose (100 mg/kg) revealed that the preventive and interventional groups exhibited markedly greater improvements in the pole and wire hang tests compared with the therapeutic group, with the interventional group demonstrating the most pronounced enhancement. This difference implies that RRJCP administration prior to the establishment of irreversible neural circuit damage induced by MPTP toxicity (i.e., during the preventive or interventional stages) may more efficiently preserve motor coordination by maintaining synaptic plasticity and the functional integrity of the neuromuscular junction. In contrast, once significant behavioral deficits have manifested during the therapeutic stage, a higher dose is required to achieve an equivalent degree of recovery. Collectively, the behavioral improvements not only provide intuitive functional evidence for the neuroprotective effects of RRJCP but also preliminarily underscore the temporal advantage conferred by early intervention.

Histopathological results revealed the morphological basis of protection. HE staining showed that MPTP caused disorganized arrangement, reduced numbers, and pyknotic necrosis of neurons in the substantia nigra pars compacta. This finding is consistent with previous studies. After RRJCP treatment, the neuronal morphology was largely restored to normal, with more orderly arrangement and reduced nuclear pyknosis. This result visually confirmed that RRJCP could mitigate the direct toxic damage of MPTP to substantia nigra neurons, protecting neuronal survival and structural integrity. More importantly, TH immunohistochemistry and Western blot results further localized this protective effect to dopaminergic neurons. TH is the rate-limiting enzyme for dopamine synthesis, and its expression levels directly reflect the quantity and functional state of dopaminergic neurons [30]. In this study, MPTP induced the loss of dopaminergic neurons in the midbrain substantia nigra. RRJCP treatment significantly increased the area of TH-positive neurons in the substantia nigra and the expression levels of TH protein, clearly demonstrating its antagonistic effect on the core pathological aspect of PD-the loss of midbrain substantia nigra dopaminergic neurons. This provides a solid cellular foundation for the observed behavioral improvements. Notably, irrespective of whether administration occurred during the preventive, interventional, or therapeutic phases, high-dose RRJCP consistently reversed MPTP-induced downregulation of TH expression, suggesting that its mechanism of action may encompass a dual attribute: providing reparative support for already compromised neurons while concurrently exerting preemptive defensive effects on intact neurons. Further analysis of the medium-dose (100 mg/kg) effects revealed that both the preventive and therapeutic groups exhibited superior restoration of TH protein expression compared with the interventional group. This discrepancy implies that preventive pre-administration may confer a state of “preconditioning” tolerance upon dopaminergic neurons against subsequent neurotoxin exposure, thereby enabling effective neuroprotection at a relatively lower dose.

Oxidative stress is considered an early and persistent event in the pathological cascade of PD [31]. The neurotoxin MPTP is a classic model for studying PD mechanisms, as it can selectively induce pathological and behavioral manifestations in primates that closely resemble those of human PD [32,33]. MPTP itself is a protoxin, converted in the brain by monoamine oxidase B (MAO-B) in astrocytes into the toxic cation MPP^+^. MPP^+^ is then specifically taken up into DA neurons via the high-affinity dopamine transporter (DAT), where it inhibits complex I activity in mitochondria, blocking oxidative phosphorylation and ultimately leading to energy depletion, exacerbated oxidative stress, and apoptosis [34,35]. This study measured serum levels of MDA, SOD, and GSH-PX. After MPTP modeling, the oxidative damage marker MDA content increased significantly, while the activities of the antioxidant defense markers SOD and GSH-PX decreased significantly, consistent with previous studies. RRJCP treatment effectively reversed this trend, reducing MDA levels and increasing SOD and GSH-PX activities. This finding not only confirms the potent direct or indirect antioxidant capacity of RRJCP but also directly links its protective effects to a key pathogenic mechanism of PD-redox imbalance. Reducing oxidative stress helps maintain mitochondrial function, reduce protein misfolding, and inhibit activation of apoptotic signaling pathways. Notably, high-dose RRJCP consistently ameliorated oxidative stress markers irrespective of the timing of administration, indicating that its antioxidant efficacy demonstrates robust efficacy regardless of timing. Further analysis of the medium-dose (100 mg/kg) effects revealed that the interventional group exhibited superior reduction in MDA levels compared with both the preventive and therapeutic groups. This discrepancy implies that concurrent administration of RRJCP during MPTP exposure enables its active constituents to exert immediate and efficient blockade of oxidative damage at the peak window of oxidative insult, potentially through direct scavenging of MPP^+^-induced free radicals or chelation of pro-oxidant metal ions. In contrast, pre-activation of antioxidant enzyme systems during the preventive phase and oxidative damage repair during the therapeutic phase may require higher doses to achieve comparable suppression of lipid peroxidation.

The pathological mechanisms of Parkinson’s disease are complex, involving mitochondrial dysfunction, oxidative stress, neuroinflammation, and apoptosis, among other processes. This study combined traditional in vivo phenotypic analysis with modern network pharmacology prediction, providing a clear roadmap for elucidating the multi-target mechanisms of RRJCP. Core targets (such as AKT1, EGFR, BCL2) and significantly enriched pathways (such as the PI3K-AKT signaling pathway) identified through network analysis guided the direction of subsequent Western blot validation. The PI3K/AKT pathway is a key signaling pathway regulating cell survival, proliferation, and apoptosis, and its function is often inhibited in neurodegenerative diseases [36]. Activated AKT can inhibit multiple pro-apoptotic factors such as Bad and GSK-3β through phosphorylation while promoting pro-survival pathways such as mTOR, ultimately stabilizing the mitochondrial membrane and inhibiting Caspase activation [37,38]. At the mechanistic level, there is a close causal link between the inhibition of oxidative stress and apoptosis. The observed reduction in MDA and elevation of SOD and GSH-PX in this study indicated a decrease in intracellular reactive oxygen species levels and an enhancement of the antioxidant defense system. This improved redox state is a key prerequisite for inhibiting mitochondrial pathway apoptosis. Studies have shown that oxidative stress can directly lead to a decrease in mitochondrial membrane potential, promoting cytochrome C release and subsequently activating the Caspase cascade, leading to apoptosis [39,40]. In this study, while reducing oxidative stress, RRJCP significantly increased the expression levels of p-PI3K, p-AKT, and Bcl-2, and decreased the levels of Bax and Cleaved Caspase-3 in MPTP-damaged substantia nigra tissue. Therefore, our data support a mechanism of action: RRJCP may directly or indirectly activate the PI3K/AKT pathway through its active components. Activation of this pathway, on the one hand, enhances cellular antioxidant responses by regulating downstream factors (such as Nrf2) (manifested as increased SOD and GSH-PX, decreased MDA), and on the other hand, inhibits the mitochondrial apoptotic pathway by modulating the balance of Bcl-2 family proteins. Ultimately, this collectively protects substantia nigra dopaminergic neurons from MPTP-induced toxic damage, achieving comprehensive improvements in behavioral, pathological, and biochemical indicators. Notably, the molecular effects elicited by the medium dose (100 mg/kg) exhibited phase-specific divergence: the preventive group displayed more pronounced activation of p-AKT and suppression of Cleaved Caspase-3; the interventional group showed greater efficacy in upregulating p-PI3K; whereas the therapeutic group achieved significant improvement solely in TH expression. This differential molecular response profile suggests that preventive pre-administration may enhance the threshold of neuronal resistance to subsequent apoptotic stimuli through pre-activation of downstream AKT survival signaling. During the interventional phase, concurrent administration of RRJCP and MPTP may preferentially trigger PI3K-mediated membrane-proximal signaling events to counteract acute toxicity. In the therapeutic phase, however, the efficiency of reactivating key signaling cascades is limited, necessitating a higher dose to reconstitute a fully integrated survival signaling network. Collectively, these findings underscore the unique value of preventive strategies at the molecular level: by pre-emptively initiating the phosphorylation cascade of the PI3K/AKT pathway, a robust endogenous defense barrier can be established prior to the onset of neurotoxic insult, thereby fundamentally reducing the probability of apoptotic execution pathway activation. This provides mechanistic rationale supporting early intervention with RRJCP in populations at elevated risk for PD.

In the present study, the neuroprotective effects of RRJCP were evaluated in an MPTP-induced chronic PD mouse model across three administration paradigms—preventive, interventional, and therapeutic—revealing a potential advantage of early intervention, particularly preventive administration. By integrating network pharmacology predictions with in vivo experimental validation, the involvement of the PI3K/AKT signaling pathway in RRJCP-mediated antioxidant and anti-apoptotic effects was examined, providing experimental reference for the application of medicine–food homologous resources in neurodegenerative diseases. Regarding phytochemical characterization, LC-MS analysis with stringent spectral filtering tentatively identified 25 compounds in RRJCP, spanning flavonoids, phenolic acids, triterpenoids, organic acids, and amino acids, among which 15 were consistent with previous reports, whereas the remaining 10 compounds, although tentatively assigned by spectral library matching, have not been previously documented with authentic standard verification in *Rosa roxburghii* fruit or juice. Nevertheless, RRJCP is a multi-component extract, and it remains unclear whether its neuroprotective effects derive from a single active constituent or a synergistic interplay among multiple components. Although prior studies have reported that certain compound classes present in *Rosa roxburghii* exhibit antioxidant or neuroprotective potential, the present study did not perform activity validation on isolated monomers; thus, the observed pharmacodynamic effects cannot be attributed to specific chemical entities. These issues require further elucidation through subsequent activity-guided fractionation and monomer validation studies. Furthermore, the mechanistic investigation herein was primarily focused on the PI3K/AKT pathway, and whether RRJCP engages additional signaling networks implicated in PD pathogenesis warrants further exploration. Finally, notable discrepancies persist between animal models and human PD regarding pathological progression and clinical manifestations, and the translational potential of RRJCP requires further validation in preclinical and clinical settings. Future studies incorporating organoid models or patient-derived cells, along with comprehensive dose–effect and long-term toxicity assessments, will be essential to advance the application of RRJCP as a neuroprotective functional food or adjunctive therapeutic agent. Furthermore, the genus Rosa encompasses a wide variety of species with distinct phytochemical profiles. Future studies comparing the neuroprotective efficacy of RRJCP with extracts from closely related species, such as Rosa rugosa or Rosa canina, would be valuable for elucidating the structure-activity relationships and species-specificity of the observed effects.

In summary, this investigation integrated behavioral assessments, biochemical assays, histopathological evaluations, molecular biology techniques, and bioinformatics analyses to comprehensively characterize the neuroprotective profile of RRJCP in an MPTP-induced murine model of PD. The underlying mechanistic basis appears to involve, at least in part, activation of the PI3K/AKT signaling cascade, attenuation of oxidative damage, and suppression of apoptotic cell death. These observations extend the prospective utility of RRJCP in the field of neuroprotection and provide additional experimental substantiation and conceptual foundations for advancing natural product-derived interventions against PD. Owing to its status as a medicinal-edible resource, RRJCP exhibits favorable safety profiles and broad accessibility, highlighting its potential as both a prophylactic and an adjunctive therapeutic regimen for Parkinson’s disease.

## 4. Materials and Methods

### 4.1. Animals

A total of 144 male C57BL/6 mice, 8 weeks old, weighing 18–20 g, and of specific pathogen-free (SPF) grade, were obtained from the Experimental Animal Center of Guizhou Medical University. The mice were housed in an SPF-classified animal facility affiliated with the Natural Products Research Center of Guizhou Province, featuring a barrier-sustained environment and individually ventilated cages. This experimental site holds license SYXK (Qian) 2018-0001. In accordance with the ethical standards set forth in the Chinese Guidelines for the Care and Use of Laboratory Animals, the present study received institutional approval (No. 2400109) from the Animal Ethics Committee at Guizhou Medical University, ensuring full procedural compliance.

### 4.2. RRJCP Sample Preparation

Guizhou Peihua Company (Guiyang, China) supplied the RRT juice utilized in this investigation. The RRT juice was evaporated and concentrated through rotating evaporation and vacuum freeze-drying. Finally, the RRT juice concentrate powder was stored at 4 °C in sterilized glass bottles [27].

### 4.3. LC-MS/MS Analysis of RRJCP

RRJCP was dissolved in 80% methanol to a final concentration of 10 mg/mL. After vortexing and ultrasonication, the mixture was centrifuged, and the supernatant was filtered through a 0.22 μm membrane. Analysis was performed using a Dionex Ultimate 3000 RSLC liquid chromatography system (Thermo Fisher Scientific, Sunnyvale, CA, USA) coupled with a Thermo Scientific Q Exactive Focus high-resolution mass spectrometer (Thermo Fisher Scientific, Waltham, MA, USA). Chromatographic separation was achieved on a Waters Acquity UPLC HSS T3 column (100 mm × 2.1 mm, 1.8 μm) (Waters Corporation, Milford, MA, USA) maintained at 40 °C. The mobile phase consisted of 0.1% formic acid in water (A) and 0.1% formic acid in acetonitrile (B), delivered at a flow rate of 0.3 mL/min. The gradient elution program was as follows: 0–5 min, 5% B; 5–42 min, 5% → 95% B; 42–47 min, 95% B; 47.1–50 min, 5% B. Mass spectrometric detection was carried out using a HESI-II source operated in positive/negative ion switching mode. Source parameters were set as follows: spray voltage, 3.0 kV (+)/2.5 kV (−); capillary temperature, 320 °C; sheath gas, 35 arb; auxiliary gas, 10 arb; probe heater temperature, 350 °C; and S-Lens RF level, 60. Data were acquired in Full MS-ddMS^2^ mode. Full MS scans were performed over an *m*/*z* range of 100–1500 at a resolution of 70,000, with an AGC target of 1 × 10^6^ and a maximum injection time of 100 ms. MS/MS scans were acquired at a resolution of 17,500, with an AGC target of 2 × 10^5^ and a maximum injection time of 50 ms. The isolation window was set to 1.5 *m*/*z*, and stepped normalized collision energies of 20, 40, and 60 were applied. Dynamic exclusion was set to 5 s. Raw data were processed using Compound Discoverer software (version 3.3), and compound identification was performed by matching accurate mass, retention time, and fragmentation patterns against spectral libraries.

### 4.4. Mouse Model

Comprising 144 male C57BL/6 mice, the experimental population underwent random division into three main groups. (prevention, intervention, treatment; n = 48), each containing six subgroups (n = 8). A chronic MPTP (Shanghai yuanye Bio-Technology Co., Ltd., Shanghai, China) model was induced by 10 injections over 5 weeks (25 mg/kg MPTP, preceded by 250 mg/kg probenecid (MedChemExpress, Monmouth Junction, NJ, USA) 30 min earlier). RRJCP (50, 100, 200 mg/kg) or Madopar (Mad) (Shandong Xinhua Pharmaceutical Co., Ltd., Zibo, China) (50 mg/kg) was administered by gavage. Ctrl groups received equivalent volumes of saline and/or DMSO. Prevention Group: Different doses of RRJCP, Mad, or saline were administered by gavage for 5 weeks, followed by 5 weeks of probenecid/MPTP injections The normal control subgroup received DMSO + saline injections. Intervention Group: Different doses of RRJCP, Mad, or saline were administered by gavage for 5 weeks, concurrent with 5 weeks of probenecid/MPTP injections The normal control subgroup received concurrent DMSO + saline injections. Treatment Group: Probenecid/MPTP injections were administered for 5 weeks to establish the model first, followed by 5 weeks of gavage administration with different doses of RRJCP, Mad, or saline. The normal control subgroup received saline by gavage for 5 weeks first, followed by DMSO + saline injections for 5 weeks (Figure 1A). Figure 1A created with BioGDP.com [41].

### 4.5. Behavioral Tests

Open field test: After drug administration was completed, the open field test was conducted on all mice. Individual subjects were positioned within an open field apparatus measuring 60 cm × 60 cm × 60 cm, and their locomotor activity was videotaped for a 10 min interval via the Test Video 4 system. Between successive trials, the arena was thoroughly cleaned with 75% ethanol to remove olfactory traces deposited by prior occupants. Post hoc quantification of aggregate travel distance for each experimental group was performed using EthoVision XT 17 software.

Pole test: After drug administration, the pole test was performed. Construction of the apparatus involved a wooden pole measuring 50 cm in height and 10 mm in diameter, covered with gauze to improve grip, with a small sphere of 2.5 cm in diameter mounted at the summit. Animals were situated with forepaws contacting the sphere and the head directed superiorly. Chronometric recording captured the temporal interval for descent from the spherical apex to the basal platform. Triplicate trials were conducted for each mouse, and the arithmetic mean of the latencies on the pole was recorded as the final value.

Wire Hang test: In the Wire Hang test, a 5 mm diameter steel wire was fixed horizontally. Each mouse was allowed to grip the wire with its two forepaws, and whether it could also grasp the wire with its hind paws was observed. Grasping behavior was quantified via a tiered scoring system: bilateral hind paw contact yielded three points, unilateral hind paw contact yielded two points, and absence of grip yielded one point. Assessment occurred in triplicate for each animal, with inter-trial intervals maintained at or exceeding 10 min. Aggregation of individual scores and calculation of mean values preceded statistical analysis.

### 4.6. Antioxidant Indexes Measurements

Upon termination of behavioral procedures, all mice entered a 12 h fast with continuous water provision. Anesthesia was achieved using pentobarbital sodium (4%, 50 mg/kg) administered intraperitoneally, facilitating blood withdrawal from the orbital sinus. Specimens were centrifuged at 2000× *g* for 20 min to separate the serum. The serum levels of MDA, SOD, and GSH-PX (Nanjing Jiancheng Biotechnology Research Institute Co., Ltd., Nanjing, China) were measured according to the respective commercial assay kits’ instructions.

### 4.7. Hematoxylin and Eosin Staining

Harvested cerebral tissues underwent primary fixation in 4% paraformaldehyde. Ethanol gradient ascent (70%, 80%, 90%, 100%) achieved dehydration enroute to paraffin processing and microtome sectioning. Sections were dewaxed with xylene and rehydrated through a descending ethanol gradient (100%, 90%, 80%, 70%) before immersion in distilled water. Chromogenic visualization employed hematoxylin, bluing, eosin counterstain, dehydration, and neutral balsam preservation. Terminal pathological assessment and photomicrography utilized optical microscopy.

### 4.8. Immunohistochemical Staining

Paraffin sections underwent antigen retrieval via heat induction in citrate buffer (pH 6.0, 20 min), with natural cooling to room temperature. Endogenous peroxidase activity was quenched using 3% hydrogen peroxide (10 min, room temperature), prior to goat serum blocking (20 min). Overnight incubation with primary TH antibody (1:50, 4 °C) (Servicebio, Wuhan, China)was followed by PBS washing and secondary antibody application (37 °C, 30 min). DAB substrate enabled colorimetric detection, and hematoxylin counterstaining (3 min). Dehydration through ascending ethanol grades (75%, 85%, 95%, 100%) and xylene clearing (10 min each) preceded air drying and neutral resin mounting. Quantitative assessment of TH positive area employed ImageJ (Fiji) software (version 1.54f).

### 4.9. Identification of Active Constituents and Target Prediction in RRJCP

Based on prior investigations conducted by our research group, a total of 25 distinct chemical constituents within RRJCP were characterized utilizing liquid chromatography-mass spectrometry (LC-MS) [41]. To identify bioactive candidates, these compounds were subsequently filtered through the Traditional Chinese Medicine Systems Pharmacology database and analysis platform (TCMSP, https://www.tcmsp-e.com/, accessed on 27 January 2025). The screening parameters were established with a threshold of oral bioavailability (OB) ≥ 30% combined with a drug-likeness (DL) index ≥ 0.18. For those ingredients meeting the specified criteria, their corresponding Simplified Molecular Input Line Entry System (SMILES) notations were retrieved from the PubChem repository (https://pubchem.ncbi.nlm.nih.gov/, accessed on 27 January 2025). The acquired SMILES strings were then submitted to the Swiss Target Prediction web server (http://www.swisstargetprediction.ch/, accessed on 27 January 2025) to perform reverse docking-based target inference. Following the consolidation and removal of redundant entries from the predicted targets, a comprehensive spectrum of potential therapeutic targets associated with the active components of RRJCP was established. Concurrently, a collection of targets implicated in the pathology of Parkinson’s disease was sourced by querying the GeneCards (https://www.genecards.org/, accessed on 27 January 2025) and OMIM databases (https://www.omim.org/, accessed on 27 January 2025) using “Parkinson disease” as the keyword. The search outputs derived from both platforms were merged, and overlapping entries were eliminated, thereby yielding a definitive set of potential therapeutic targets relevant to Parkinson’s disease.

### 4.10. Construction of Protein–Protein Interaction Network

The overlapping targets shared between the bioactive constituents of RRJCP and the pathological targets implicated in Parkinson’s disease were determined using the VENNY 2.1.0 online tool (https://bioinfogp.cnb.csic.es/tools/venny/, accessed on 27 January 2025). A Venn diagram was subsequently plotted to visualize the intersection, thereby delineating the prospective therapeutic targets through which RRJCP may exert its anti-PD effects. The set of intersecting genes was then submitted to the STRING database (https://cn.string-db.org/, accessed on 27 January 2025) to construct a protein–protein interaction (PPI) network. During this process, isolated nodes lacking any interactions were removed from the analysis. Finally, the resulting network data were exported and imported into Cytoscape version 3.10.3 for graphical rendering and subsequent topological interrogation.

### 4.11. Gene Ontology (GO) Analysis and Kyoto Encyclopedia of Genes and Genomes (KEGG) Pathway

To elucidate the biological significance of the overlapping gene set shared between RRJCP active components and Parkinson’s disease pathology, Gene Ontology (GO) annotation and Kyoto Encyclopedia of Genes and Genomes (KEGG) pathway enrichment assessments were conducted using the DAVID functional annotation suite (https://davidbioinformatics.nih.gov/summary.jsp, accessed on 27 January 2025). Specifically, GO analysis was employed to characterize the involvement of these intersecting genes across three distinct categories: biological processes (BP), cellular components (CC), and molecular functions (MF). Subsequent interpretation of the KEGG enrichment data facilitated the identification of pivotal molecular pathways governing essential physiological activities. Ultimately, the resulting GO terms and KEGG enrichment outputs were transferred to the online bioinformatics visualization platform (http://www.bioinformatics.com.cn/, accessed on 27 January 2025) for graphical representation and further in-depth examination.

### 4.12. Western Blotting

Homogenization of substantia nigra specimens in RIPA lysis buffer containing 1% PMSF (Beyotime, Shanghai, China) utilized a tissue grinder to generate approximately 10% homogenate. Post homogenate ice incubation (30 min) preceded centrifugation at 12,000 rpm, 4 °C, 10 min in a high speed refrigerated centrifuge, yielding supernatant for EP tube transfer and total protein extraction. BCA (Servicebio, Wuhan, China) quantification determined loading buffer volume. Heat denaturation (100 °C, 10 min, metal bath) was followed by ambient cooling and −80 °C storage. Prepared SDS PAGE gel placement in electrophoresis chamber preceded sample boiling (100 °C, 10 min) and mixing. Electrophoretic separation occurred initially at 80 V (30 min), then 120 V (60 min). Transfer onto PVDF membrane utilized 220 mA constant current for 1.5 h. Subsequent processing comprised 5% BSA blocking, TBST washing, and primary antibody overnight incubation (4 °C). (TH, 1:2000, Proteintech; Bax, 1:2000, Proteintech; Bcl-2, 1:2000, Proteintech; Caspase-3, 1:2000, Proteintech; Cleaved-Caspase-3, 1:2000, Proteintech; PI3K, 1:2000, Proteintech p-PI3K, 1:2000, Proteintech; AKT, 1:2000, Proteintech; p-AKT, 1:2000, Proteintech, Wuhan, China; GAPDH, 1:10,000, HUABIO; β-actin, 1:10,000, HUABIO, Hangzhou, China). The next day, the membrane was washed with TBST, incubated with a diluted secondary antibody on a horizontal shaker at room temperature for 120 min, and washed again with TBST. Finally, chemiluminescent detection was performed in a darkroom. The exposed protein bands were imported into ImageJ (Fiji) software for grayscale value analysis.

### 4.13. Statistical Analysis

All data underwent statistical analysis via Microsoft Excel and GraphPad Prism 10.1.2. Results are reported as arithmetic mean ± SD. One-way ANOVA served for group comparisons, and *p* values less than 0.05 indicated statistical significance.

## 5. Conclusions

In this study, RRJCP exerted protective effects against MPTP-induced Parkinson’s disease model mice by improving behavioral impairments, alleviating brain tissue damage, reducing oxidative damage, inhibiting apoptosis, and activating the PI3K/AKT pathway. This study provides a scientific basis for the application of RRJCP, a medicine–food homology resource, in the protection against Parkinson’s disease, and offers preclinical evidence supporting its development as a functional food or adjuvant therapeutic agent with neuroprotective properties.

## Figures and Tables

**Table 1:** Identification of Chemical Ingredients in RRJCP.

No.	Compound Name	Molecular Formula	RT (min)	Measured *m*/*z*	Adduct	ppm	Diagnostic Fragments (*m*/*z*)	mzVault Score	Peak Area
1	(+)-Catechin hydrate [19]	C_15_H_14_O_6_	9.275	289.07178	M−H	0.07	245.08, 203.07, 109.03	96.3	1.70 × 10^9^
2	Isoquercitrin [20]	C_21_H_20_O_12_	13.743	463.08792	M−H	−0.61	300.03, 271.02, 255.03	95.6	1.45 × 10^7^
3	Ellagic acid [19]	C_14_H_6_O_8_	13.461	300.99884	M−H	−0.50	229.01, 185.02, 145.03	95.1	4.41 × 10^8^
4	Cianidanol *	C_15_H_14_O_6_	11.392	291.08640	M+H	0.31	139.04, 123.04, 147.04	94.7	9.20 × 10^6^
5	Quinic acid [20]	C_7_H_12_O_6_	0.858	191.05527	M−H	−4.40	191.06, 85.03, 93.03	94.5	5.54 × 10^7^
6	Naringenin chalcone *	C_15_H_12_O_5_	13.199	273.07562	M+H	−0.48	153.02, 147.04, 119.05	94.4	3.24 × 10^6^
7	Citric acid [20]	C_6_H_8_O_7_	0.968	191.01888	M−H	−4.45	111.01, 87.01, 85.03	93.8	9.30 × 10^7^
8	Ferulic acid [21]	C_10_H_10_O_4_	10.896	195.06516	M+H	−0.15	177.05, 145.03, 117.03	93.2	5.44 × 10^6^
9	Sucrose [22]	C_12_H_22_O_11_	0.882	341.10852	M−H	−1.23	341.11, 89.02, 59.01	92.4	1.13 × 10^7^
10	L-Leucine [20]	C_6_H_13_NO_2_	1.530	132.10194	M+H	0.23	86.10, 69.07, 132.10	92.1	5.71 × 10^7^
11	Procyanidin B1 [20]	C_30_H_26_O_12_	8.827	577.13495	M−H	−0.35	407.08, 289.07, 125.02	91.8	6.61 × 10^8^
12	L-Tryptophan [20]	C_11_H_12_N_2_O_2_	5.247	205.09723	M+H	0.39	188.07, 146.06, 118.07	91.4	3.20 × 10^7^
13	Morin [23]	C_15_H_10_O_7_	17.772	301.03519	M−H	−0.63	151.00, 107.01, 121.03	90.8	4.81 × 10^6^
14	Corilagin [24]	C_27_H_22_O_18_	10.202	633.07330	M−H	−0.06	301.00, 275.02, 229.01	90.5	1.11 × 10^8^
15	L-Arginine [20]	C_6_H_14_N_4_O_2_	0.988	175.11903	M+H	−2.68	70.07, 60.06, 116.07	90.4	2.18 × 10^7^
16	Lindenenol *	C_15_H_18_O_2_	13.255	231.13808	M+H−H_2_O	−0.48	213.13, 69.03, 195.12	88.1	2.53 × 10^7^
17	Ligustilide *	C_12_H_14_O_2_	14.238	191.10669	M+H	0.16	145.10, 105.07, 173.10	87.9	2.21 × 10^7^
18	Emodin *	C_15_H_10_O_5_	12.154	271.05981	M+H	−1.07	271.06, 69.00, 229.05	86.7	9.87 × 10^6^
19	p-Coumaric acid [20]	C_9_H_8_O_3_	9.870	165.05473	M+H	0.67	147.04, 119.05, 91.05	86.4	4.38 × 10^7^
20	Quillaic acid *	C_30_H_46_O_5_	18.579	487.34210	M+H-H_2_O	0.18	487.25, 119.09, 107.09	86.3	6.36 × 10^6^
21	Oleanonic acid *	C_30_H_46_O_3_	23.513	455.35223	M+H	−0.55	189.16, 95.09, 119.09	85.8	3.28 × 10^6^
22	Tetrahydroxyxanthone *	C_13_H_8_O_6_	23.570	259.02460	M−H	−0.81	259.02, 215.03, 187.04	81.8	2.98 × 10^6^
23	Asiatic acid *	C_30_H_48_O_5_	19.586	489.35721	M+H	−0.49	205.16, 201.16, 187.15	80.7	3.21 × 10^7^
24	Geraniin *	C_41_H_28_O_27_	7.919	951.07471	M−H	0.20	301.00, 275.02, 229.01	78.9	1.41 × 10^8^
25	Rosamultin [24]	C_36_H_58_O_10_	19.603	685.37183	M−H	−0.25	487.34, 425.34, 407.33	78.0	6.41 × 10^7^

* No literature has confirmed.

## Data Availability

The original contributions presented in this study are included in the Article and Supplementary Materials. Further inquiries can be directed to the corresponding authors.

## Supplementary Materials

The following supporting information can be downloaded at: https://www.mdpi.com/article/10.3390/ph19050711/s1, Table S1: Ingredients in RRJCP by LC-MS.

## Abbreviations

The following abbreviations are used in this manuscript:

AKT	Protein kinase B
Bcl-2	B-cell lymphoma 2
Bax	BCL2-associated X protein
Caspase-3	Cysteine-dependent aspartate-specific protease-3
GSH-PX	Glutathione peroxidase
HE	Hematoxylin-eosin
IHC	Immunohistochemistry
MDA	Malondialdehyde
MHF	Medicine and food homology
RRT	*Rosa roxburghii* Tratt
RRJCP	*Rosa roxburghii* Tratt juice concentrate powder
SOD	Superoxide dismutase
PD	Parkinson’s Disease
PI3K	Phosphoinositide 3-kinase
TH	Tyrosine hydroxylase
