# Supplementary material for: Neuroprotective Effects of Rosa roxburghii Tratt Juice Concentrate Powder in Parkinson’s Disease Mice via the PI3K/AKT Signaling Pathway
Source: Pharmaceuticals (Basel). 2026 Apr 30;19(5):711. doi: 10.3390/ph19050711 (PMC13209371; doi:10.3390/ph19050711)
Supplement: Supplementary file 1 [file pharmaceuticals-19-00711-s001.zip › pharmaceuticals-4247341-supplementary.pdf]

Supplementary material

Supplementary Table 1: Ingredients in RRTE by LC-MS

No.	Rt(min)	Ingredients	Molecular Formula	Molecular Weight
1	0.217	p-Hydroxybenzaldehyde	C7 H6 O2	122.0355
2	0.732	3-[(Carboxycarbonyl)amino]-L-alanine	C5 H8 N2 O5	193.973
3	0.858	Quinic acid	C7 H12 O6	192.0625
4	0.882	Sucrose	C12 H22 O11	342.1158
5	0.968	Citric acid	C6 H8 O7	192.0261
6	1.53	L-Leucine	C6 H13 N O2	131.0947
7	1.78	Maleic acid	C4 H4 O4	116.0097
8	2.048	Gallic acid	C7 H6 O5	170.0205
9	2.558	L-Phenylalanine	C9 H11 N O2	148.0524
10	5.247	L-Tryptophan	C11 H12 N2 O2	204.09
11	7.919	Geraniin	C41 H28 O27	952.0819
12	8.36	Corilagin	C27 H22 O18	634.0806
13	8.523	Eriodictyol	C15 H12 O6	288.0632
14	8.611	Procyanidin B2	C30 H26 O12	278.0807
15	8.827	Procyanidin B1	C30 H26 O12	578.1422
16	9.275	(+)-Catechin hydrate (+)-	C15 H14 O6	290.0791
17	9.294	Protocatechualdehyde	C7 H6 O3	138.0318
18	9.296	Benzoic acid	C7 H6 O2	105.0103
19	9.396	Abscisic acid	C15 H20 O4	264.1363
20	9.527	Peucedanol	C14 H16 O5	264.0979
21	9.87	p-Coumaric acid	C9 H8 O3	164.0474
22	9.877	Bilobalide	C15 H18 O8	326.1002
23	10.895	7-Methoxycoumarin	C10 H8 O3	144.0211
24	10.896	Ferulic acid	C10 H10 O4	194.0579
25	11.267	Procyanidin A2	C30 H24 O12	576.1268
26	11.358	Kaempferitrin	C27 H30 O14	578.1634
27	11.392	Cianidanol	C15 H14 O6	290.0791
28	11.437	Isomucronulatol 7-O-glucoside	C23 H28 O10	442.1838
29	11.898	Hyperoside	C21 H20 O12	464.0945
30	11.905	Nodakenin	C20 H24 O9	376.1132
31	11.967	Prim-O-glucosylcimifugin	C22 H28 O11	486.171
32	12.154	Emodin	C15 H10 O5	270.0525
33	13.199	Naringenin chalcone	C15 H12 O5	272.0684
34	13.255	Lindenenol	C15 H18 O2	248.1413
35	13.461	Ellagic acid	C14 H6 O8	302.0061
36	13.542	Fraxin	C16 H18 O10	392.0719
37	13.569	Lobetyolin	C20 H28 O8	442.1836
38	13.738	Morin	C15 H10 O7	302.0425
39	13.743	Isoquercitrin	C21 H20 O12	464.0953

40	13.778	Ginkgolide B	C20 H24 O10	407.1078
41	13.86	7-Methoxy-4-methylcoumarin	C11 H10 O3	190.0631
42	13.951	Ethyl caffeate	C11 H12 O4	93.0458
43	14.119	Arglabin	C15 H18 O3	264.1362
44	14.238	Ligustilide	C12 H14 O2	190.0994
45	14.241	α-Asarone	C12 H16 O3	208.1094
46	15.04	Perillene	C10 H14 O	118.0783
47	15.77	Linderalactone	C15 H16 O3	262.1204
48	16.411	Astringin	C20 H22 O9	406.126
49	17.606	5-O-Methylvisammioside	C22 H28 O10	452.1663
50	17.996	o-Veratraldehyde	C9 H10 O3	184.0735
51	18.062	18 β-Glycyrrhetintic Acid	C30 H46 O4	488.3501
52	18.121	Rhaponticin	C21 H24 O9	398.1576
53	18.143	Atractyloside A	C21 H36 O10	494.2361
54	18.579	Quillaic acid	C30 H46 O5	504.3451
55	18.653	Dehydrocostus lactone	C15 H18 O2	248.1414
56	19.193	Glabrolide	C30 H44 O4	486.3348
57	19.26	L-Kawain	C14 H14 O3	230.0943
58	19.502	Ethyl ferulate	C12 H14 O4	222.0886
59	19.516	Medicagenic acid	C30 H46 O6	240.174
60	19.586	Asiatic acid	C30 H48 O5	488.3498
61	19.586	Pedunculoside	C36 H58 O10	696.4084
62	19.603	Rosamultin	C36 H58 O10	686.3791
63	20.55	Polygalic acid	C29 H44 O6	488.3137
64	21.264	Rutaevin	C26 H30 O9	486.187
65	21.854	Limonin	C26 H30 O8	470.1934
66	23.513	Oleanonic acid	C30 H46 O3	454.345
67	23.57	Tetrahydroxyxanthone	C13 H8 O6	260.0319
68	24.944	Pinosylvin	C14 H12 O2	212.0837
69	26.56	Bayogenin	C30 H48 O5	488.3499
70	49.261	Caffeic acid	C9 H8 O4	180.0414
71	49.753	3,5-Dimethoxy-4-hydroxybenzaldehyde	C9 H10 O4	182.0579
